# Networks of pre-diagnostic circulating RNA in testicular germ cell tumour

**DOI:** 10.1038/s41598-024-84484-z

**Published:** 2025-01-14

**Authors:** Joshua Burton, Trine B. Rounge, Trine B. Haugen, Marcin W. Wojewodzic

**Affiliations:** 1 Department of Life Sciences and Health, OsloMet – Oslo Metropolitan University, Oslo, Norway; 2 Department of Pharmacy, University of Oslo, Oslo, Norway; 3 Department of Research, Cancer Registry of Norway, Norwegian Institute of Public Health, Oslo, Norway; 4 Department of Chemical Toxicology, Norwegian Institute of Public Health, Oslo, Norway

**Keywords:** Network analysis, Pre-diagnostic, RNA, Testicular cancer, TGCT, WGCNA, Network topology, Cancer, Testicular cancer, Urological cancer, Systems biology, Regulatory networks

## Abstract

Testicular germ cell tumour (TGCT) is a malignancy with known inherited risk factors, affecting young men. We have previously identified several hundred differentially abundant circulating RNAs in pre-diagnostic serum from TGCT cases compared to healthy controls. In this study, we performed Weighted Gene Co-expression Network Analysis (WGCNA) on mRNA and miRNA data from these samples. Central genes (hub genes) enriched functional pathways, and regulatory feature prediction were identified for all TGCT subtypes together and according to histology. The TGCT susceptibility genes *TEX14*, *NARS2*, and *G3BP2*, were identified as hub genes in both seminoma and non-seminoma networks. We also identified *UBCA1*,* RCC1*,* FMR1*,* OAS3*, and *UBE2W* as hub genes associated with TGCT. The genes *OAS3* and *UBE2W* have previously been associated with testicular dysgenesis. Furthermore, network module analysis indicated transcription factors for oestrogen-related receptors to have a potential role during development of TGCT. The overlap between mRNA network hub genes and TGCT susceptibility genes indicates a common role in TGCT development.

**Supplementary Information:**

The online version contains supplementary material available at 10.1038/s41598-024-84484-z.

## Introduction

Testicular germ cell tumour (TGCT) is the most common form of testicular cancer, affecting primarily younger males. Over the last decades, there has been an increasing incidence of TGCT in developed countries, now levelling off in high incidence countries such as Norway and Denmark^1–4^. It is generally agreed that TGCT develops from a pre-malignancy, germ cell neoplasia in situ (GCNIS), originating through a loss of differentiation of gonocytes during foetal life, which progress to a malignancy during postnatal puberty^5^. It has been reported so far more than 94 risk loci for TGCT as well as environmental risk factors, and ethnicity also affects the likelihood of developing TGCT^6–10^. TGCT is among disorders commonly associated with the so-called testicular dysgenesis syndrome (TDS), which also includes cryptorchidism, poor semen quality, and hypospadias^11,12^. However, the aetiology behind TGCT is still mainly unknown.

Advances in TGCT treatment have led to an increased 5-year survival rate, with some countries having a higher than 95% survival rate^2^, however, survivors of TGCT are still at risk of secondary cancers, cardiovascular disease, epigenetic alterations, and reduced longevity associated with cisplatin treatment^13–16^. It is therefore important to diagnose TGCT early to reduce the rounds and dosage of cisplatin-based treatment needed and to potentially reduce the long-term side effects.

Analyses of pre-diagnostic samples may increase the understanding of the progression of TGCT. Our previous studies of pre-diagnostic serum samples from the Janus Serum Bank^17,18^, identified genes associated with the development of lung cancer and TGCT^19,20^. In these studies, we used traditional linear models with negative binomial distribution employed on RNA-Seq count data. A total of 818 circulating RNAs were found to be differentially expressed in pre-diagnostic TGCT cases when compared to controls. For instance, differences in expression were observed for the male fertility-related gene, *TEX101*, and the X-chromosome inactivation gene, *TSIX* (18). To advance the field where only limited pre-diagnostic samples are available, novel methods for analysing TGCT data are required.

Weighted Gene Co-expression Network Analysis (WGCNA) is a systems biology approach that allows for increased comprehension of multidimensional transcriptomic datasets^21^. WGCNA identifies gene co-expression patterns in multiple samples from various states. WGCNA can be used to identify elements such as biomarkers or future therapeutic targets as well as screening datasets for networks of genes relating to certain traits^22^. WGCNA also allows for the comparison of such networks to show nodes and clusters that are preserved between differing states, such as histological classes. This is one of the major differences between standard RNA-Seq approaches, where linear models that assume independence of genes are used (i.e. limma, edgeR, or DESeq2)^23–25^. WGCNA does not only consider the differentially expressed genes, but also utilise the comprehensive gene list to search for inter-connectivity between clusters of genes^21^. Using these methods, previous research has found related genes to specific cancers through mRNA and miRNA analyses, including to lung adenocarcinoma ^26^, bladder cancer^27^, and breast cancer^28^.

The WGCNA can be used to obtain new information about the mechanisms underlying TGCT development. Changes in networks could indicate presence of disease or its early development^29,30^. Observed alterations within gene networks also have the potential to be used for biomarker discovery^31^. Extensive networks relying on multiple different biological components, such as transcription factors, mRNAs, miRNAs, miRNA targets, have been demonstrated in multiple diseases, including TGCT^32^. The data in this work to Mallik and coworkers originated from post-diagnostic samples of seminoma and non-seminoma and compared TGCT cases without controls. In this work, subtype-specific networks were constructed, and mRNAs, miRNA, and transcription factors were identified. In both the seminoma and non-seminoma modules, the top miRNAs were onco-related miRNAs for several types of cancers. Common hub genes, *SP1* and *MYC*, were also identified between the networks constructed for seminoma and non-seminoma^32^. Post-diagnostic TGCT networks contained Phosphatase and Tensin Homolog (*PTEN*), targeted by three miRNAs, hsa-miR-141, hsa-miR-222, and hsa-miR-21, with hsa-miR-222 also regulating KIT and Tumor Protein 53 (*TP53*)^33^. Studies which only use differential expression provide limited information of these networks, therefore a more in-depth method of comparing disease states should be pursued to obtain a more complete and comprehensive understanding of what drives TGCT development.

We aimed to identify mRNA and miRNA networks associated with TGCT development in pre-diagnostic serum samples by using WGCNA. We compared networks from both the two TGCT histological classes: seminoma and non-seminoma. We hypothesised that by using network analysis, we could propose genes and mechanisms related to TGCT development.

## Materials and methods

### Data and sample description

Samples and data are described in detail in our previous study of pre-diagnostic RNA profile in serum from TGCT patients^19^. In brief, the serum samples in the Janus Serum Bank were collected between 1972 and 2004 from participants in health surveys and Red Cross blood donors. These were linked to registry data from the Cancer Registry of Norway to identify cases and controls. We included patients who developed TGCT up to 10 years after sample donation and control samples who were cancer-free within 10 years after sample donation, matched on age at donation and sampling time. In total, 79 TGCT cases were identified, and of these, 52 were seminoma and 27 non-seminoma subtypes. These subtypes were confirmed by registry data from the Cancer Registry of Norway. In addition, 111 matched controls were retrieved. RNA profiles were generated by extracting RNAs from 400 µl serum, and small RNA sequencing libraries were produced with NEBNext kit (Cat. No E7300, New England Biolabs Inc.) and sequenced on the HiSeq 2500 (Illumina) as previously described^34^. All details for bioinformatic pre-processing procedure of raw data were published^34^, and further information can be found in the online Appendix 1.

### WGCNA pipeline steps to module preservation analysis

**Fig. 1:**
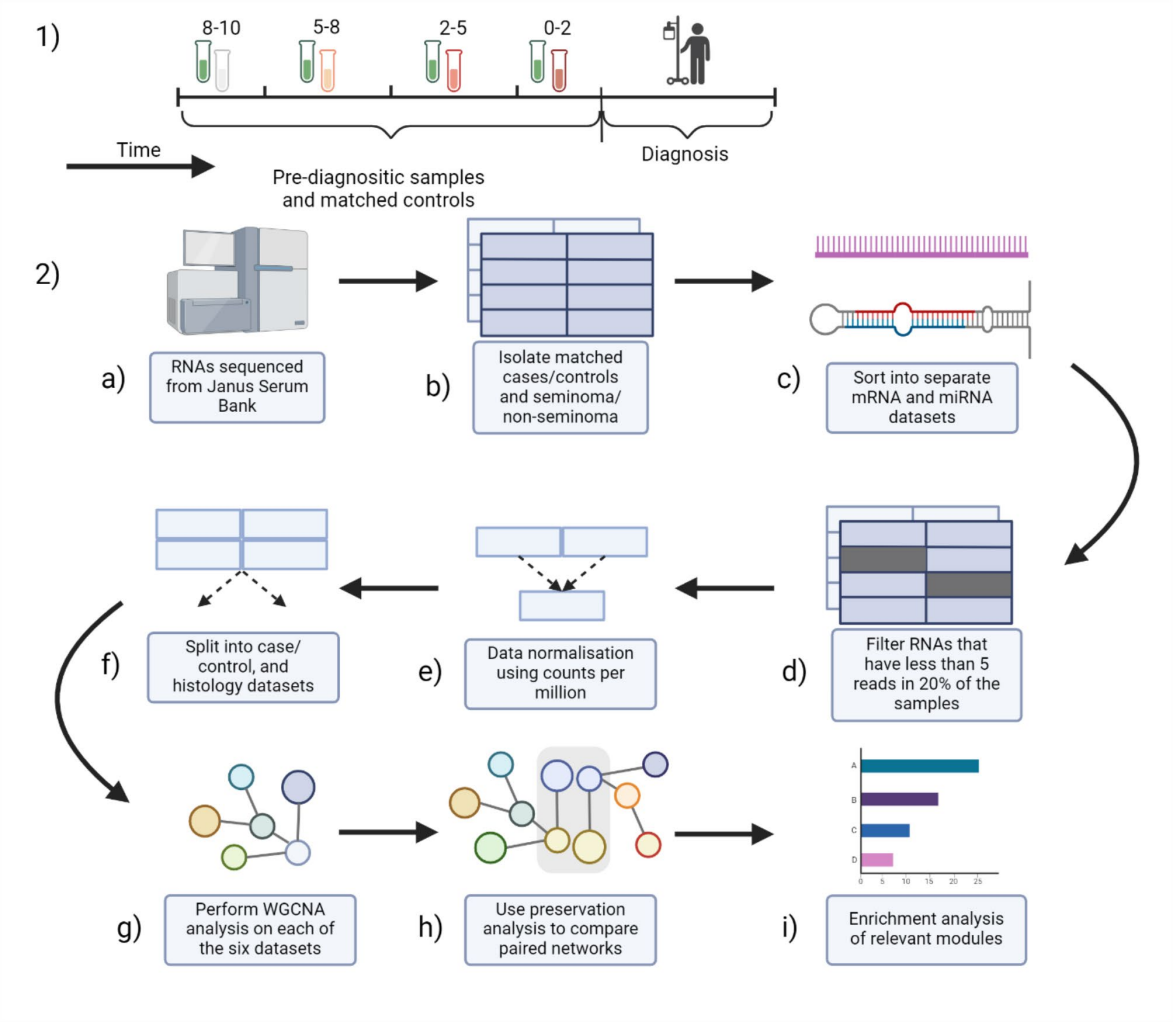
(1) Design of the study. Samples were identified from the Janus Serum Bank (JSB) in a 10-year period prior to diagnosis. (2) Workflow for analysis. (**a**) RNAs were initially sequenced from JSB serum sample, and sncRNAs were quantified. (**b**) Samples were split based on cancer diagnosis and histology and matched with controls. (**c**) mRNA and miRNA datasets were separated for different analyses. (**d**, **e** and **f**) Quality control was performed on the datasets, and data was prepared for network analyses. (**g**) WGCNA pipelines were used to perform network analyses. (**h**) The preservation analyses were performed on comparable networks. (**i**) Enrichment analysis was performed on relevant modules. Figure was created with BioRender.com.

Weighted Gene Co-expression Network Analysis (WGCNA) was employed to analyse our pre-processed and normalized count data (Appendix 1) using WGCNA R package (1.70-3). After pre-processing the data (Fig. 1), we constructed networks based on the correlations between features (mRNA or miRNA expression). These networks identify clusters of tightly interconnected correlated genes, referred as modules. We summarized each module using a highly connected hub feature called ‘eigengenes’ or ‘eigenmirs’ for mRNA and miRNA networks, respectively. Each module was assigned a specific colour. Grey modules were removed from downstream analysis as they contained genes they could not be fitted into the network analysis main modules. Additionally, we divided the TGCT samples into seminoma and non-seminoma categories and performed network construction using only mRNA counts, as there were not sufficient miRNAs available for network analysis in each histology.

### Preservation network analysis

Network preservation analysis was used to compare and assess the modules present in two different networks. This analysis identified which modules were stable (preserved) across the networks and new elements that are specific to the condition. We conducted several comparisons between different groups; (i) mRNA serum levels in cases versus controls, (ii) miRNA serum levels in cases versus controls, and (iii) mRNA serum levels between seminoma and non-seminoma. Each of these subsets had its own network construction, while ensuring a similar structure. To quantify module preservation, we used modulePreservation function with 200 permutations, implemented in WGCNA. This function measures how well the density and connectivity patterns of modules identified in a reference dataset (control samples) are preserved in a test dataset (TGCT samples) and ensure that connection found are not random. Comparing network adjacencies provides more accurate estimates of module preservation compared to standard techniques. Our code was further developed from previously published resources^35^. In addition, we performed sensitivity analysis using the ComBat function in the R package SVA (v. 3.32.1) to check batch affects and ensure the robustness of our network (Appendix 2).

### Pathway analysis

Functional annotation was performed using the enrichment analysis tool Enrichr with default settings^36–38^, by inputting gene lists from modules generated by WGCNA. Of particular interest were modules that were not preserved between cases and controls and those which were preserved between seminoma and non-seminoma. Enrichr databases were then reviewed for significantly enriched pathways and pathways associated with TGCT related conditions.

### Regulatory patterns

The regulatory patterns of the mRNAs included in the modules were investigated using a promoter motif analysis tool i-cisTarget (gbiomed.kuleuven.be/apps/lcb/i-cisTarget). A full analysis with standard parameters was run with gene symbols as the input and RefSeq r45 and v.6 of the i-cisTarget database, with only transcription factor binding site databases included in the analysis. This analysis reveals if the module is regulated by specific transcription factors for genes related to male fertility or cancer development.

### Determining miRNA targets on mRNA

New modules predicting miRNA as preserved between cases and controls underwent a miRNA prediction analysis to determine which mRNAs the preserved miRNAs targeted. Translating miRNA to mRNA targets also allows for easier comparison between the miRNA and mRNA networks. miRNA targets are determined through sequence matching. Targets were extracted from miRDB (v5.0) with a cut off score of > 70, allowing only targets of high confidence to be included in the analysis.

### Enrichment analyses and visualisation

Enrichment analyses were performed on the network and module data, firstly, KEGG^39–41^ analysis was performed, using Enrichr on the top ten hub genes in each of the mRNA cases, seminoma, and non-seminoma modules. A parallel enrichment analysis was also performed on all the genes in the module to give an overall prediction for enriched pathways. For miRNA cases, mRNA targets were used for enrichment analysis, with the cut off score of > 70. Cytoscape (v. 3.9.0) was used to visualise the network modules. Cytoscape files were created using the export function in WGCNA, generating both the node and the edge files necessary to visualise.

### Assessing possible clinical relevance of eigengenes for seminoma

To assess the possible clinical relevance of identified eigengenes associated with seminoma, we have evaluated the associations between the expression of *SOX17* and *KIT*, which are known markers for seminoma, and new genes that were discovered as part of our analysis on seminomas. To do so, we first normalised first raw counts using a variance stabilizing transformation from DeSeq2 (version 3.20), and then we constructed linear models between consecutive genes and *SOX17* and *KIT*.

## Results

### Modules for mRNAs for cases and controls

Modules with TGCT-related genes in mRNA cases include the black_(Cases)_, magenta_(Cases)_, purple_(Cases)_, salmon_(Cases)_, and the midnightblue_(Cases)_ modules (Table 1). Main characteristics of the networks were presented in a supplementary material (Appendix 3).

**Table 1:** mRNA network modules specific to mRNA cases when compared to mRNA controls.

Module	Eigengenes			Top KEGG 2021 Pathway for HUB genes	Top KEGG 2021 Pathway for all genes in module
	E1	E2	E3		
**Black**	*UBAC1*	*UBE2W*	*OAS3*	**-**	**-**
Cyan	*KCNB1*	*CRAT*	*NUDT3*	Citrate cycle	-
Greenyellow	*GHRHR*	*CTNND1*	*STXBP4*	-	-
Grey60	*OPRD1*	*ZBTB2*	*MME*	Renin-angiotensin system	Pentose and glucose interconversions
Lightcyan	*PCDHGB6*	*PCDHGA10*	*PCDHGA9*	-	Ubiquitin mediated proteolysis
Lightgreen	*DNAJC17*	*RP11-77K12.1*	*PFN2*	Nicotinate and nicotinamide metabolism	Thiamine metabolism
Lightyellow	*XPOT*	*SLC38A11*	*PLD1*	SNARE interactions in vesicular transport	beta-Alanine metabolism
**Magenta**	*BEST3*	*SLC16A2*	*RCC1*	**-**	**AMPK signaling Pathway**
**Midnightblue**	*RTCB*	*JAKMIP1*	*MAP3K12*	**Taste transduction**	**Phenylalanine, tyrosine and tryptophan biosynthesis**
**Purple**	*CPNE5*	*FMR1*	*RBFOX2*	**-**	**Notch signaling pathway**
**Salmon**	*KIAA2026*	*IGF2BP3*	*SBF2*	**-**	**-**

Top three eigengenes in each module listed, as well as significant enrichment analysis results for top 10 eigengenes and for all genes included in the module. Rows in bold contain genes of interest within the top three eigengenes (E1, E2, E3). Additional information about mean, normalized expression of eigengenes E1 can be found in Supplementary Table 3.

Preservation analysis performed on the mRNA case/control networks showed 11 new modules, i.e. modules that were present in cases but absent from controls (Table 2). New modules are of particular interest as they show important genes involved in TGCT development. Seven preserved modules with a significant number of mRNAs that are shared between paired modules were identified. Finally, one of the modules had several mRNAs shared between paired modules but not to a significant degree, known here as non-preserved modules.

**Table 2:** mRNA network modules for non-seminoma, with preservation state shows mRNA non-seminoma vs. seminoma analysis results.

Module	Eigengenes			Top KEGG 2021 Pathway for Top 10 HUB genes	Top KEGG 2021 Pathway for All Genes in Module	Preservation state
	E1	E2	E3			
Black	*CRAT*	*SLC6A9*	*KCNB1*	Glycosaminoglycan biosynthesis	-	Preserved (68)
**Blue**	***LMBR1***	***NPFFR2***	***STPG2***	**-**	**-**	**Preserved (549)**
Brown	*IL12RB1*	*HMGXB4*	*CHRNA1*	-	-	Preserved (40)
Cyan	*NREP*	*GFRAL*	*ELMOD3*	-	-	New
Darkgreen	*ALMS1*	*CWF19L2*	*KIF18A*	-	Ascorbate and aldarate metabolism	Preserved (6)
Darkgrey	*PRKACB*	*ANKRD13A*	*MARK1*	-	-	Preserved (11)
Darkorange	*C6orf118*	*HBG2*	*HBE1*	-	-	New
Darkred	*CBWD2*	*LRSAM1*	*EVC*	Nicotinate and nicotinamide metabolism	-	Preserved (21)
Darkturquoise	*PPP1R1C*	*GPR141*	*RASIP1*	-	-	Preserved (8)
Green	*MAF*	*AGRN*	*LGMN*	-	Dilated cardiomyopathy	Preserved (181)
Greenyellow	*OPRD1*	*ZBTB2*	*TRIM72*	-	-	Preserved (44)
Grey60	*PELI1*	*TEC*	*PDE6A*	Nitrogen metabolism	-	Preserved (20)
Lightcyan	*LZIC*	*ZER1*	*ZNF670-ZNF695*	Valine, leucine and isoleucine degradation	-	Preserved (12)
Lightgreen	*ARID4A*	*TMEM57*	*DSTYK*	Arginine and proline metabolism	-	New
**Lightyellow**	***ANAPC4***	***SCGN***	***KCND3***	**-**	**Various types of N-glycan biosynthesis**	**New**
Magenta	*AAMDC*	*RYR2*	*GLDN*	-	N-Glycan biosynthesis	Preserved (115)
**Midnightblue**	***ULK2***	***OGFOD1***	***SPG11***	**-**	**-**	**New**
Orange	*CENPI*	*CSMD2*	*TMEM161B*	Pentose phosphate pathway	-	New
Pink	*DPCD*	*RACK1*	*VWA5B2*	-	Ferroptosis	Preserved (12)
Purple	*DNMT3A*	*DET1*	*PANK3*	Pantothenate and CoA biosynthesis	-	Preserved (173)
Red	*JAKMIP1*	*MAP3K12*	*RTCB*	-	-	Preserved (114)
Royalblue	*RABEPK*	*CCM2*	*GRAMD1C*	-	-	Preserved (9)
Salmon	*WAS*	*CFHR3*	*SCUBE1*	-	Non-small cell lung cancer	Preserved (17)
Tan	*DEK*	*AC002310.13*	*AUH*	-	-	Preserved (10)
Turquoise	*SHISA5*	*RP11-164J13.1*	*ARIH2*	Circadian rhythm	Amphetamine addiction	Preserved (151)
Yellow	*CROT*	*HELB*	*PRELID3A*	Aminoacyl-tRNA biosynthesis	-	Preserved (32)

Included are the top three hub genes in each module as well as enrichment analysis results for all 10 hub genes and for all genes included in the module. Modules that were preserved show number of genes preserved between seminoma and non-seminoma. Rows in bold contain genes of interest within the eigengenes (E1, E2, E3). Additional information about mean, normalized expression of eigengenes E1 can be found in Supplementary Table 5.

Blue_(Cases)_ showed significant preservation, with 603 preserved genes, and a p-value < 0.001. The module midnightblue_(Cases)_ was identified as being a new module, as well as black_(Cases)_, magenta_(Cases)_, purple_(Cases)_, and salmon_(Cases)_ modules.

### Modules for mRNA for seminoma and non-seminoma

The modules of interest for TGCT development in the seminoma (SE) network were the blue_(SE)_, lightyellow_(SE)_, and midnightblue_(SE)_. The modules of interest in the non-seminoma (NS) network were yellow_(NS)_ and red_(NS)_.

Analysis of histology-related networks identified several modules of interest that were preserved between seminoma and non-seminoma. Of the 22 modules in the seminoma network and the 26 in the non-seminoma network, 20 were preserved between the two. One such module that was significantly preserved was the blue_(SE)/_red_(NS)_ module, which shared 114 genes between both histology classes. There were also two new modules present for seminoma and six new modules present for non-seminoma, showing possible histology-specific genes of interest. Of note is the recurring presence of this module between all case networks. We also found significant associations between *SOX17* with *PANK3* and *TBX5* (*p* < 0.05) but not for *KIT*. The results are reported in the Supplementary Tables 7–8 and Supplementary Fig. 1.

### Modules for miRNAs for cases and controls

The module of interest within miRNA cases was the brown_(miCases)_ module.

Preservation analysis of miRNA networks identified four modules that were preserved between cases and controls and one new module, specific to cases. mRNA target prediction for the top three eigenmiRs within this module was performed using > 70 as the cut off value for prediction score (Sup. Table 1). A total of 1310 unique mRNA targets were identified. EigenmiRs targeting prediction showed that all three eigengenes were predicted to target *MAP3K* genes as well as related pathway genes. Hsa-miR-30e-5p was predicted to target *KRAS* and other members of the RAS oncogene family, as well the gene *MAP3K12*, with a confidence score of 78.

### Enrichment analyses

KEGG analysis of the mRNA cases network showed enrichment of the Ubiquitin mediated proteolysis pathway in lightcyan_(Cases)_. Purple_(Cases)_ showed significant enrichment of the Notch signalling pathway. Magenta_(Cases)_, a new module, showed significant enrichment of the AMPK signalling pathway. Finally, the preserved green_(Cases)_ module also showed significant enrichment of pathways in cancer (Table 1). Furthermore, the red_(SE)_ module showed significant enrichment of the T cell receptor signalling pathway (Table 3). Salmon_(NS)_, a preserved module between seminoma and non-seminoma, showed significant enrichment of the non-small cell lung cancer associated pathway (Table 2).

**Table 3:** mRNA network modules for seminoma, with preservation state shows mRNA seminoma vs. non-seminoma analysis results.

Module	Eigengenes			Top KEGG 2021 Pathway for Top 10 HUB genes	Top KEGG 2021 Pathway for All Genes in Module	Preservation state
	E1	E2	E3			
Black	*USP14*	*GHRHR*	*CTNND1*	-	-	Preserved (21)
Blue	*UBE2W*	*OAS3*	*TNFAIP2*	-	-	Preserved (114)
Brown	*CFAP100*	*TTC7B*	*NRCAM*	-	-	Preserved (181)
Cyan	*RP1-27O5.3*	*PSEN1*	*CERKL*	-	-	Preserved (26)
Darkgreen	*NLRP11*	*DNAJB14*	*ADCY7*	-	-	Preserved (12)
Darkred	*HEPH*	*HMG20B*	*ZNF565*	-	-	Preserved (6)
Green	*DPPA2*	*PUDP*	*TMPRSS6*	-	Ascorbate and aldarate metabolism	Preserved (44)
Greenyellow	*GLIPR1*	*TBX18*	*ITPKB*	-	-	Preserved (40)
Grey60	*FAM151B*	*EEA1*	*RAD23B*	-	-	Preserved (32)
Lightcyan	*FASN*	*SCN2B*	*C1orf198*	-	-	Preserved (8)
Lightgreen	*PPP1R13B*	*UBXN8*	*ZNF280C*	-	-	New
Lightyellow	*UVSSA*	*KLHL21*	*FIGNL1*	-	-	New
Magenta	*SCN5A*	*CLUH*	*ST8SIA1*	-	-	Preserved (115)
Midnightblue	*ZC4H2*	*ATP1B1*	*GRID2*	-	-	Preserved (17)
Pink	*TBX5*	*HSPA9*	*EIF4G1*	-	Shigellosis	Preserved (68)
Purple	*RP11-729L2.2*	*SMAD4*	*PNPLA7*	Viral myocarditis	-	Preserved (11)
**Red**	***BEST3***	***SLC16A2***	***CAMK1D***	**-**	**T cell receptor signalling pathway**	**Preserved (151)**
Royalblue	*RAB8B*	*ZNF143*	*POM121C*	-	-	Preserved (10)
Salmon	*SHC3*	*UGGT1*	*IARS2*	-	-	Preserved (9)
Tan	*PDE6D*	*CENPN*	*LARP4*	-	-	Preserved (20)
Turquoise	*CHRNA7*	*NECTIN3*	*PPP1R9A*	-	-	Preserved (549)
**Yellow**	***PANK3***	***PANK2***	***COPZ1***	**Pantothenate and CoA biosynthesis**	**Hippo signalling pathway**	**Preserved (173)**

Included are the top three eigengenes in each module as well as enrichment analysis results for top 10 eigengenes and for all genes included in the module. Modules that were preserved show number of genes preserved between seminoma and non-seminoma. Rows in bold contain genes of interest within the eigengenes (E1, E2, E3). Additional information about mean, normalized expression of eigengenes E1 can be found in Supplementary Table 4.

Pathway enrichment analysis was then performed using the eigenmiR’s mRNA targets. The following KEGG pathways showed significant enrichment in the brown_(miCases)_ module: long term potentiation, axon guidance, regulation of the actin cytoskeleton, ErbB signalling pathway, T cell receptor signalling pathway, neurotrophin signalling pathway, MAPK signalling pathway, choline metabolism in cancer, FoxO signalling pathway, glioma, cGMP-PKG signalling pathway, renal cell carcinoma, cellular senescence, and B cell receptor signalling pathway. A further enrichment analysis was performed on the top ten eigenmiRs in brown_(miCases)_, within results from the database Jensen COMPARTMENTS, showing significant enrichment of the following entries: extracellular exosome complex, lysosomal multienzyme complex, microvesicle, EmrE multidrug transporter complex, PTEN phosphatase complex, Phosphatidylinositol phosphate phosphatase complex, and apoptotic body.

### Network figures

We used Cytoscape to explore further network characteristics. Midnightblue_(SE)_ consisted of 12 genes, including nine of the eigengenes as well as *TEX14*, *ABCB1*, and *DARS2*. Based on an enrichment analysis on these 12 genes, the DisGeNET database showed an association (adjusted p value < 0.1, OR = 114.02) with “Carcinoma testes” indicating a potential link to TGCT. Additionally, we observed enrichment (p value < 0.05) in male fertility-related disorders, including male infertility, decreased testis weight, and abnormal spermatocyte morphology, according to the MGI Mammalian Phenotype Level 4 2021 database.

### Modules with shared eigengenes

**Fig. 2:**
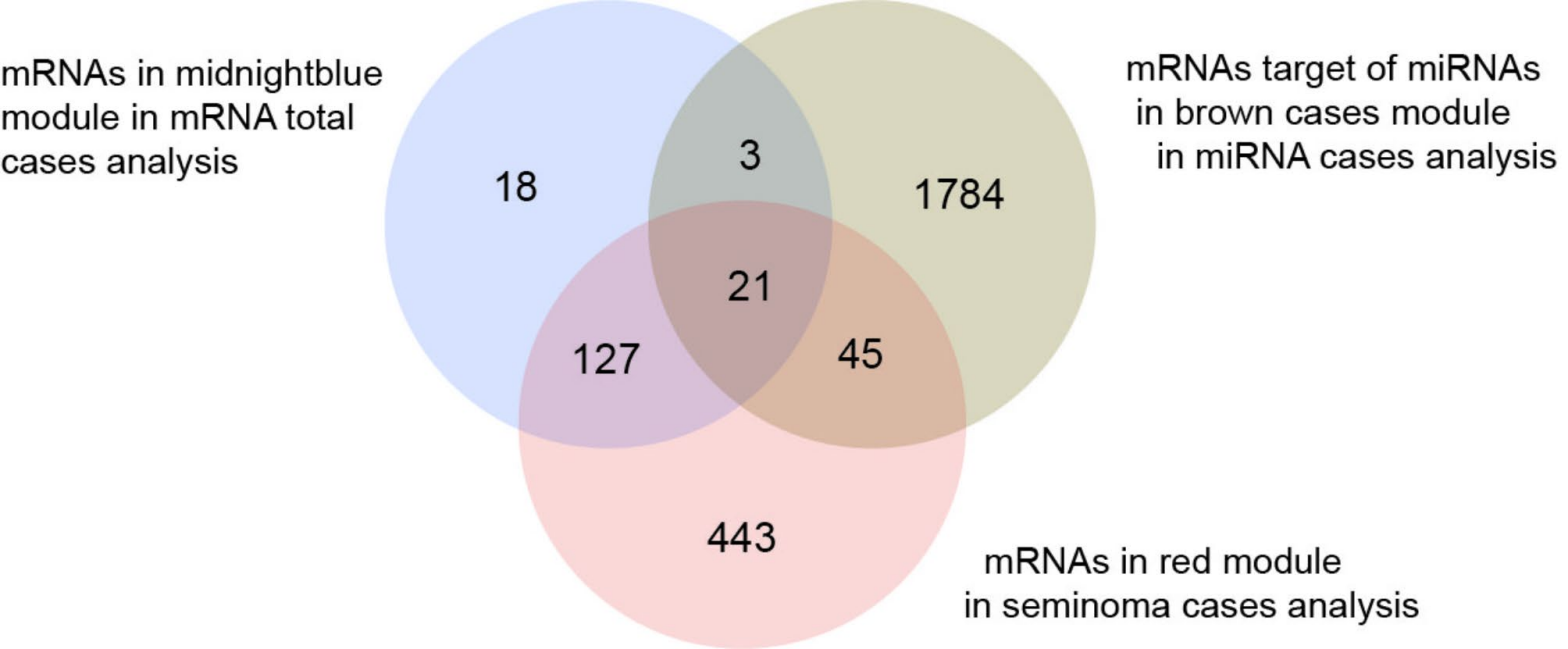
Common genes between each of the three modules containing either the *MAP3K12* mRNA or miRNAs that are known to target the *MAP3K12* gene with a confidence score of 70 in miRbase. These modules were midnightblue_(Cases)_, brown_(miCases)_ and red_(NS)_. Common genes between these modules were analysed due to the presence of similar eigengenes.

Analysis of the eigengenes in modules revealed a pattern emerging in most networks related to TGCT cases. The repeated appearance of the gene *MAP3K12* within the eigengenes, and the presence of MAPK signalling pathways within enrichment analysis were further investigated by isolating the modules in which this appeared and determining mRNA commonality between the three. The brown_(miCases)_ module was included through its predicted targets with the same confidence scores used previously. The 21 genes common to all modules were the following: *ATRX*,* BEND7*,* DCAF12*,* FYCO1*,* GMEB2*,* GRM3*,* ICK*,* IGLON5*,* KLHL14*,* MAP3K12*,* MAPK4*,* MASTL*,* NRIP3*,* PER2*,* PPP1R37*,* PRKCE*,* SCN3A*,* SLC25A36*,* STYX*,* TTLL2*,* UCHL5* (Fig. 2).

### Regulatory feature prediction

**Fig. 3:**
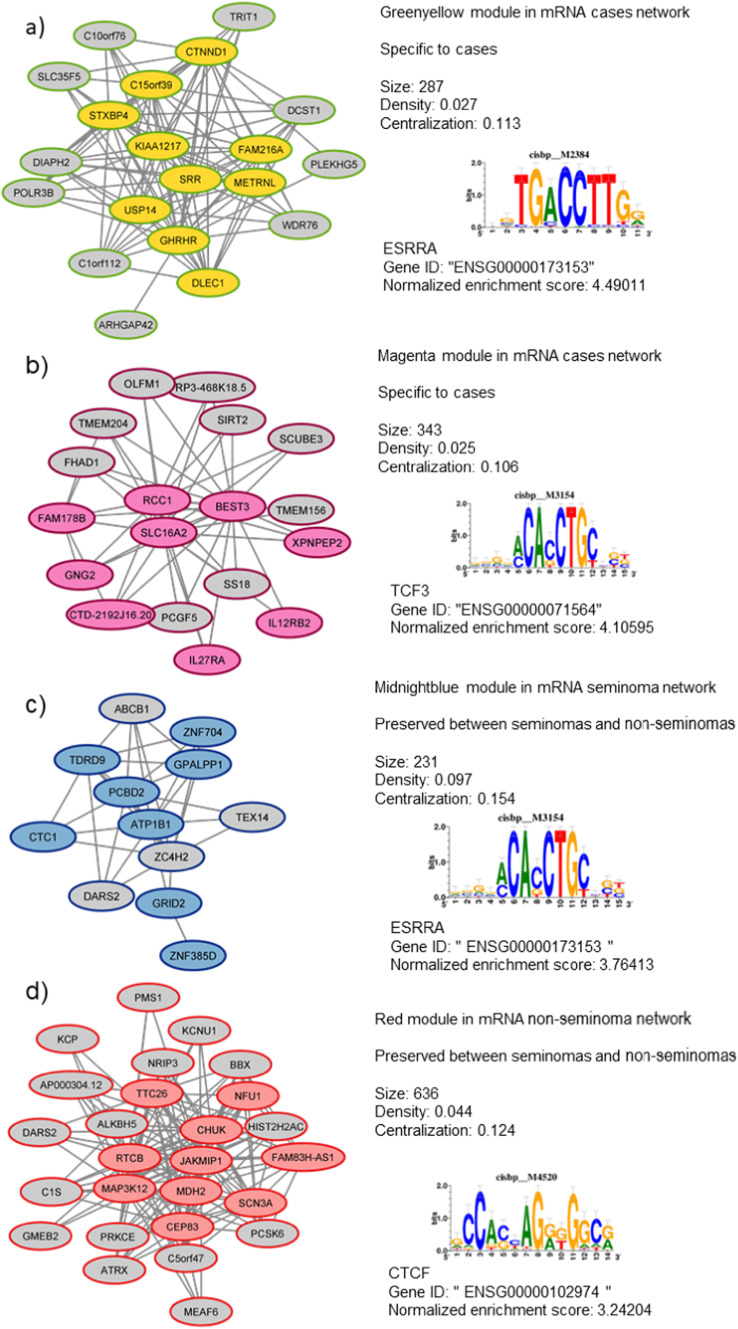
Network model of modules within mRNA from (**a**, **b**) all cases, (**c**) seminoma, and (**d**) non-seminoma. Connections between genes show a weighted correlation in expression. The distance measurement threshold for this network was set to 0.3. Genes with coloured backgrounds are eigengenes for each module. Sequence motifs show cisbp regulatory element with highest normalised enrichment score for all genes in the module. Module was selected for visualisation due to specificity to TGCT, as well the nodes passing the adjacency threshold set in the analysis.

Analysis of the genes of modules using the prediction tool i-cis Target showed regulatory elements with possible links to TGCT development. For both the modules greenyellow_(Cases)_ and midnightblue_(SE)_, the highest scoring (4.5 and 3.8, respectively) regulatory elements from the cisbp database was oestrogen-related receptor alpha gene transcription factor (*ESRRA*), with *ESRRG* also amongst the highest scoring for midnightblue_(SE)_ (Fig. 3). The highest scoring regulatory element of the red_(NS)_ module was associated with *CTCF*, and for the magenta(_Cases_) module, the highest score regulatory feature was associated with *TCF3*.

## Discussion

In our previous analysis of RNA profiles from serum samples we identified RNAs which were differentially expressed in patients prior to diagnosis of TGCT. As gene products and regulatory elements often operate in close relationships, we used network analysis framework and the tool WGCNA to identify interplay between genes and ncRNAs that could be involved in TGCT development. When comparing the networks constructed from mRNA case and mRNA control data, we were able to identify modules specific to cases that contained genes that were previously known to be associated with TGCT. As we can discern existing insights related to TGCT within the central genes of our network, we have reason to trust in the reliability of our findings. When comparing the networks constructed from mRNA case and mRNA control data, we were able to identify modules specific to cases that contained GWAS genes that were known to be associated with TGCT, indicating validity of the network analyses.

### mRNA cases network

Within the mRNA cases network there were several modules with eigengenes associated with both TGCT and male subfertility. The new black_(Cases)_ module’s top eigengene, *UBCA1*, has previously been identified as downregulated in TGCT tumour samples compared to controls^42^. This suggest that *UBCA1* is involved in early TGCT development and could be used as a potential biomarker. In addition, links to early defects in the male reproductive system of mice have been described with *UBE2W*, the second hub gene in this module^43^. In that study, knock-out mice were used to investigate the role UBE2W plays in testis development. Testis is vulnerable to the loss of UBE2W as seen through the high rate of male infertility after loss. UBE2W knock-out causes an increase in testicular vacuolation defects, pointing to its role in the maintenance of the testis structure. Defects in the male reproductive system of humans were also linked to the third hub gene, *OAS3*. Missense variants of the *OAS3* gene were found in disorders of sexual development and hypospadias, a component of the testicular dysgenesis syndrome, associated with TGCT^44^. *UBE2W* and *OAS3* were also present in the blue_(SE)_ module’s eigengenes, and *UBCA1*, *UBE2W*, and *OAS3* appeared in the red_(NS)_ module’s eigengenes. This indicates that these genes may play an important role in the formation of both seminoma and non-seminoma due to their repeated appearance as eigengenes in both the overall cases, and in both histological classes.

The eigengene *RCC1* in the new magenta_(Cases)_ module has known associations with TGCT. *RCC1* can form a fusion gene with *ABHD12B*, and this fusion, *RCC1-ABHD12B*, has been shown to be present in 9% of TGCT tissues but was not present in any control testis tissues^45^. However, another study showed expression of *RCC1-ABHD12B* in 60% of GCNIS samples, in 80% of seminoma samples, and in all embryonal carcinoma cell lines^46^. In this study, another fusion, *RCC1-HENMT1*, was also observed in a significant number of TGCT tissues of undifferentiated histological classes of TGCT, including all GCNIS samples, and all other TGCT samples, excluding on yolk sac tumour.

The MAPK signalling pathway-related gene *MAP3K12* was observed as an eigengene for the new midnightblue_(Cases)_ module. Previously *MAP3K12* has been associated with prostate cancer, and regulation of *MAP3K12* by miRNAs could suppress prostate cancer progression^47^. *MAP3K12* was also observed as one of the genes associated with activation of oncogene-induced angiogenesis in hepatocellular carcinomas^48^. MAPK signalling pathways have also been labelled as one of the dominant functional pathways in spermatogenesis, alongside the AMPK pathways^49^. We observed both MAPK-related genes and significant association with AMPK-related pathways in the new modules of the mRNA cases network. The involvement of these pathways in TGCT development should be investigated further as they may play an important role.

In addition, the new purple_(Cases)_ module eigengene *FMR1* has been investigated in TGCT patients and could be used to predict overall prognosis^50^. Expression levels of *FMR1* was shown positively correlated with the clinical outcome of TGCT and therefore could act as tumour suppressors alongside *AR* and *GPC3* genes^50^. The eigengene *RBFOX2* has previously been studied in cryptorchidism susceptibility. Paralogs of this gene are expressed in the gubernaculum, and failure of this fibrous cord to elongate during development causes the birth defect commonly associated with TGCT^51,52^.

The new salmon_(Cases)_ module contained the eigengene *IGF2BP3*, also known as *IMP3*. The IMP3 protein is an oncofoetal protein which has known roles in both embryogenesis and carcinogenesis^53^. Within teratomas, IMP3 expression has been seen in 100% of mature teratoma components and in 96% of all metastatic testicular teratomas. In this study, it was also observed to be expressed in 99% of all other TGCT components^53^. Therefore, it should also be investigated further for possible role in development of GCNIS and its progression to TGCT, due to its shared roles in embryogenesis and carcinogenesis.

### Histology-classes related networks

In general, pure seminomas comprise around 50% of TGCTs and non-seminomas approx. 30%. The remaining 20% are of mixed type, i.e. contain both seminoma and non-seminoma components^54^ Due to this heterogeneity of non-seminomas, interpretation of specific findings for non-seminomas should be interpreted with caution.

Within the seminoma network, one of the new module, the lightyellow_(SE)_, contained the eigengene *G3BP2*. Studies of TGCT have identified the cytogenetic band 4q21.1 as being a possible location for a susceptibility locus. The genes positioned within this band include *G3BP2*, whilst not fully labelled as a TGCT susceptibility gene, its location within this band should be noted. Our observation of this gene within seminoma could explain its identification within genome-wide associations studies (GWAS), and its role within seminoma histology should be investigated further.

The preserved midnightblue module_(SE)_ also contained an eigengene that has previously been identified in TGCT studies (Fig. 3). The *GRID2* gene is an extremely large gene (1.47Mbs within 4q22.3) that could be involved in hepatocellular carcinomas^55^. Focal deletions in *GRID2* have been seen in TGCT, though it has primarily been seen within non-seminoma^56^. Finally, within the non-seminoma networks, the module yellow_(NS)_ contained the eigengene *NARS2*, which has previously been identified as a TGCT susceptibility gene through GWAS within the cytogenetic band 11q14.1 ^9^ and is also involved in ovarian serous cystadenocarcinomas as a possible progression associated gene^57^.

Eigengenes within these networks, which are related to TGCT, subfertility, and disorders of sexual development are potential liquid biopsy biomarkers. The pathways associated with male reproductive health related genes in pre-diagnostic samples could be potential targets for further research, to explain the reason for their presence in serum. Although there is not one common pathway or mechanism between these eigengenes, we know that TGCT and cancer in general are multifaceted and complex. Even within histological classes of TGCT, tumour growth stems from multiple cell types with multiple mechanisms, and therefore we should expect many shared pathways for all the eigengenes related to TGCT.

TGCT analysis using network methods has been performed previously on TGCT tissue, which has allowed the identification of genes that are differentially expressed between histological classes^32^. In particular, the transcription factor-target gene pairs, *MYC* and *SP1*, were found to be common eigengenes in both seminoma and non-seminoma, as well as the miRNA miR-182-5p^32^. However, within our pre-diagnostic data, we did not see *MYC* or *SP1* as eigengenes in any of the seminoma or non-seminoma modules. We found a significant association between *PANK3* and *TBX5* with *SOX17*, suggesting a possible clinical relevance of these two novel findings. The Human Protein Atlas (proteinatlas.org) indicates that higher *PANK3* protein abundance is associated with seminoma subtypes but has lower abundance in non-seminomas (proteinatlas.org/ENSG00000120137-PANK3/cancer/testis + cancer#IHC). However, it is difficult to determine the differences between *TBX5* abundance in seminomas vs. non-seminomas in The Human Protein Atlas. In this data, medium expression has been reported in seminoma cancer patients when compared to embryonal carcinoma (proteinatlas.org/ENSG00000089225-TBX5/cancer/testis + cancer). There is no established association between the *TBX5* gene and the development of seminoma or other forms of testicular cancer. *TBX5* is a member of the T-box family of transcription factors, which plays crucial roles in various developmental processes.

### Inter-network features

The mRNAs *RTCB*, *JAKMIP1*, and *MAP3K12*, as well as the miRNA hsa-miR-30e-5p, were present as eigengenes in nearly all case related modules, and hsa-miR-30e-5p is thought to potentially target both *MAP3K12* and *RTCB*. When investigating the common mRNAs shared between all three, the presence of fertility- and cancer-related genes was observed. Early spermatid production relies on ion channels for both overall fertility and normal sperm physiology, and the gene *SCNA3* is involved in this process and defects within these channels are associated with male infertility^58^. The precise role of *ATRX* in human gonadal development is still not entirely clear, however, links between the gene *ATRX* and *DMRT1* have been previously noted^59^. ATRX acts downstream of the genes *SPY* and *SOX9*, which are crucial for Sertoli cell development and upregulation of anti-Müllerian hormone during testicular development *in utero*^59,60^. Within mice models, *styx* seems to be strongly involved in spermatid development, with ablation of *styx* in mice causing a 1,000-fold reduction in spermatozoa production^61^. These shared genes between three case-specific modules show common male fertility-related associations, indicating again the presence of possible markers for TGCT progression.

Another shared component between networks was the midnightblue_(SE)_ module. When comparing the seminoma and non-seminoma networks together, it was noted that this midnightblue module was preserved between the two histological classes. Modules that were shared between seminoma and non-seminoma could offer insights into pathways and genes of interest common to most types of TGCT. Initially, the gene *GRID2* had been identified as a gene of interest due to its associations with non-seminoma. However, when visualising the preserved midnightblue_(SE)_ module in Cytoscape (Fig. 3), further genes of interest were noted, such as the gene *TEX14*, which was associated with the hub genes for the module, though not an eigengene itself. *TEX14* has been identified as a susceptibility locus for TGCT. As shown previously, other TGCT susceptibility genes also appeared amongst the hub genes of modules in both the seminoma and non-seminoma modules, including *NARS2* in the yellow_(NS)_ module and *G3BP2* in the lightyellow_(SE)_ module. Both these modules are significantly preserved between both histological classes. Our findings are in line with the GWAS identifying these loci regardless of histology, however, our findings propose that changes at these loci occur earlier than previously thought (i.e. prior diagnosis).

In the midnightblue_(SE)_ and the greenyellow_(Cases)_ modules, we also saw the enrichment of *EGRR* genes during the regulatory elements analysis. *ERR*s are a group of genes that code for oestrogen-related receptors, and previously they have been found to be crucial in controlling energy metabolism for both normal and cancerous cells, particularly *ERRA* and *ERRG* ^62^. In some cancers, such as endometrial, colorectal, and prostate cancer, *ERR* expression is low, which suggests that *ERR*s have an overall negative effect on tumour progression^63,64^. In the formation of Leydig cells, *ERR*s are also crucial, as lack of *ERR*s has been shown to affect Leydig cell morphology and physiology in cultures^65^. *ERRA* has also shown to mediate the adaption of metabolic systems to the tumour microenvironment and could be involved in the direct activation of Wnt11/β-catenin pathway, and this in turn leads to an increase in the capacity of cells to migrate^66^. Overall, these genes are strongly involved in the molecular characteristics of Leydig cell tumours. Although this is not TGCT, the presence of *ERR* transcription factor enrichment in our seminoma samples could indicate a joint aetiology between the two tumour types.

Further analysis of regulatory elements identified transcription factors for the gene *CTCF* as being associated with the genes within the red_(NS)_ module. *CTCF* and its paralogues have been previously identified in testicular tumour cells. It has been suggested that the co-expression of *CTCF* and *BORIS* could be responsible for epigenetic deregulation leading to cancer. Furthermore, the mechanisms surrounding *CTCF/BORIS* could be an essential factor leading to the immortalisation of testicular cancer cells^66^.

### Strengths and limitations

We have performed analysis on unique pre-diagnostic samples and compared them to the control samples. The strength of WGCNA is the reduction in the complexity of the data, allowing for a more accurate interpretation. With this approach we have detected TGCT-related genes in addition to those previously identified with linear models. The size and scope of the dataset is also advantageous and allows us to investigate the period before TGCT diagnosis, whilst also considering confounding factors from health surveys by matching cases and controls based on these factors.

Nonetheless, sample size is a limitation of this study. This dataset contains count data for various RNA types. Whilst we were able to perform a network analysis for different histological classes on the mRNA from our data, the scarcity of other RNA types limited sub-analysis on seminoma and non-seminoma for the other types, such as miRNA. There were also two new modules present for seminoma and six new modules present for non-seminoma, showing possible histology-specific genes of interest, however, since 20% of the non-seminoma may consist of both seminoma and non-seminoma components, these finding should be interpreted with caution. An increase in statistical power would be beneficial for the WGCNA analyses across histological classes as well as alternate RNA types.

## Conclusion

We identified TGCT-related genes with high degrees of connectivity in TGCT exclusive networks, such as *TEX14*, *NARS2*, and *G3BP2*. In addition, we observed the enrichment of cancer-related mismatch repair KEGG pathway and have identified predicted transcriptional factors such as oestrogen-related receptors (*ERR*s) in multiple TGCT modules. Enrichment analysis also showed “Carcinoma testes” and several male fertility-related disorders as significantly enriched within a seminoma module.

Finally, we show the potential for RNAs discussed here to be utilised as biomarkers for earlier detection of TGCT. These genes of interest (eigengenes) should be validated independently in other cohorts and/or be evaluated mechanistically in relevant cell lines using for instance CRISPR-Cas-9 technology. The pathological relevance of the validated hub genes needs to be further examined. Systematic investigation of these genes and their roles in cell lines would allow for validation of the altered phenotypes for each of these genes and propose a possible order of molecular events during tumorigenesis. We believe that our findings have the potential to increase the understanding of the aetiology of TGCT.

## Electronic supplementary material

Below is the link to the electronic supplementary material.

Supplementary Material 1

## Data Availability

The datasets generated for this article are not readily available because of the principles and conditions set out in articles 6 (1) I and 9 (2) (j) of the General Data Protection Regulation (GDPR) and Norwegian Law. However, requests to access the datasets should be directed to the corresponding author and will be facilitated after complying with Ethical Permission and Norwegian Law.
