# Supplementary material for: Networks of pre-diagnostic circulating RNA in testicular germ cell tumour
Source: Sci Rep. 2025 Jan 14;15:1910. doi: 10.1038/s41598-024-84484-z (PMC11733264; doi:10.1038/s41598-024-84484-z)
Supplement: Supplementary file 1 — Supplementary Material 1 [file 41598_2024_84484_MOESM1_ESM.docx]

**Online Appendix for**

**Networks of pre-diagnostic circulating RNA in testicular germ cell tumour**

Joshua Burton^1^, Trine B. Rounge^2,3^, Trine B. Haugen^1^ and Marcin W. Wojewodzic*^3,4^

**Appendix 1**

1. ***Details for bioinformatics analysis***

Pre-processing of raw transcriptomics data was run using a high-performance computing cluster at the Cancer Registry of Norway. Detailed description can be found in a previously published article ^1^ (https://github.com/sinanugur/sncRNA-workflow). In brief, the reads generated an average depth of 18.4 million raw reads per sample. Adapters were removed with AdapterRemoval (v.2.1.7) with collapsed reads then being mapped to the human genome (hg38) with Bowtie2 (v2.2.9). Annotation set for RNAs was GENCODE (v.26) and for miRNAs was miRbase (v.22). We included mRNAs with at least five reads in more than 20% of the samples, and in the miRNA analysis we removed miRNAs that had no reads in more than 10% of the samples.

Controls and cases for the network construction were matched using age at sampling, time of sampling, and blood donation group, including separate matching for samples in the ‘0-7 years before diagnosis’ and ‘0-4 years before diagnosis’ groups as part of a sensitivity analysis (Figure 1). The same matching methods were performed between the subtypes, seminoma and non-seminoma during the network analysis based on TGCT histology. The *optmatch* R package (v0.9–11) (github.com/markmfredrickson/optmatch) was used to find these matching sets. Full overview of these procedures was given in a previous article ^2^.

Statistical analyses were performed on the processed datasets. Firstly, counts were normalised using counts per million from the EdgeR package ^3^. The R package ComBat ^4^ was then used to determine if there was any batch effect in our data. A simplified WGCNA analysis on mRNA cases data which had undergone batch correction was run. Power graph analyses were compared with the full mRNA cases analysis which had not undergone batch correction.

**Appendix 2**

***2. Sensitivity analysis***

To determine if the time to diagnosis of each sample affected network construction, a sensitivity analysis was performed by excluding the samples with a longer time period between sampling and diagnosis. As the data was split into four separate time frames from a previous study ([0-2], [2-5], [5-8], [8-10]), we first excluded the 8-10 years between sampling and diagnosis samples, which also gives us smaller datasets to work with. As a further sensitivity step, the 5-8 years between sampling and diagnosis samples were removed for a second round.

Sensitivity analysis of the mRNA networks was performed through the exclusion of samples diagnosed from 8-10 years after sampling. Weighted correlation gene networks were constructed using the remaining samples diagnosed from 0-7 years after sampling with matched controls. For the sensitivity analysis of 0-7 years, the cases network consisted of 26 modules with an average module size of 412. The largest module, turquoise_(Cases_

_0-7)_ consisted of 1948 mRNAs and had a density of 0.012 and a heterogeneity of 0.529. Control network with 8–10-year samples excluded consisted of a total of 14 modules with an average module size of 765. The largest module here was turquoise_(Controls 0-7)_ which consisted of 2200 mRNAs with a density of 0.033 and a heterogeneity of 1.324.

Enrichment analysis of 0-7 years showed several new modules in both cases and control networks. The turquoise_(Cases 0-7)_ module finds both cancer-related and germ cell development-related pathways during enrichment analysis.

Further sensitivity analysis was performed through the exclusion of all samples diagnosed 6-10 years after sampling. Weighted gene correlation networks showed that using 0–5-year samples would allow for the construction of a network with 28 modules in total. The average module size was 382.

1. ***Power calculations***

To construct a WGCNA network, a soft thresholding power must be selected to determine which RNAs are co-expressed through adjacency. This soft threshold is determined through an analysis of the network topology (the physical arrangement of nodes and connections in the network). By plotting the fit index for each power, the power value for which the scale-free topology fit index curve flattens can be visualised and used as the power value. For our networks the power value was equal to 10 for mRNA and miRNA networks.

The selection of the soft power parameter in (WGCNA) was meticulously standardised across subsequent network constructions Our primary objective was to identify an optimal soft power value, often referred to as the soft threshold, that would enable the capture of meaningful gene co-expression relationships while effectively mitigating noise and preserving the essential scale-free topology within the network. In this study, a fixed power parameter, n=10, was consistently employed for all WGCNA analyses.

To ascertain the appropriateness of the chosen soft power, we conducted a rigorous assessment of the network's scale-free topology using the scale-free topology fit index. This index was computed over a range of β values, and we deliberately selected the specific β value at which the scale-free topology fit index either plateaued or approached a value exceeding 0.8. This threshold was indicative of the successful establishment of a scale-free network structure.

It is noteworthy that the Optimal Soft Power value was uniformly set at 10 across all network construction instances. The selection of this β value was made based on its alignment with the point where the scale-free topology fit index demonstrated stabilization or achieved a notably high value.

This consistency in the choice of power parameters ensures the rigorous comparability of results across the various networks examined in this study, thereby enhancing the reliability and interpretability of our findings.

**Appendix 3**

1. **Main characteristics of the networks**

The mRNA cases network had 19 modules with a total of 10,596 mRNAs. The average module density was 0.026, and the average heterogeneity was 0.99. The mRNA control network had 11 modules with 8,462 mRNAs. The average module density was 0.028, and the average heterogeneity was 1.06. The miRNA cases network had 5 modules with 403 miRNAs. The average module density was 0.09, and the average heterogeneity was 0.52. The miRNA control network had 4 modules with 403 miRNAs. The average module density was 0.1, and the average heterogeneity was 0.49. The seminoma network had 22 modules with 11,032 mRNAs. The average module density was 0.05, and the average heterogeneity was 0.72. The non-seminoma network had 26 modules with 11,026 mRNAs. The average module density was 0.06, and the average heterogeneity was 0.72.

**Supplementary Tables**

**Supplementary table 1.** The top three eigenmiRs for the miRNA module brown_(miCases)_ with top ten highest confidence mRNA targets IDs.

| miRNA ID | Top 10 mRNA target IDs |
| --- | --- |
| hsa-miR-30e-5p | TWF1, B3GNT5, WDRZ, SCN2A, BRWD3, PTGFRN, DCUN1D3, NFAT5, KLHL20, PPARGC1B |
| hsa-miR-191-5p | NEURL4, TAF5, CREBBP, TMOD2, TJP1, MGST3, CBLN4, SPO11, INO80D, DDHD1 |
| hsa-miR-199a-3p | ADAMTSL3, KATNBL1, CELSR2, KLHL3, MAP3K4, ITGA3, LRP2, ETNK1, NID2, NAA25 |

**Supplementary table 2.** miRNA case network modules, including the top three hub miRNAs in each module as well as enrichment analysis results for top mRNA targets for the top three hub miRNAs in the module. Preservation state shows miRNA cases vs controls preservation analysis results. Modules that were preserved show number of genes preserved between miRNA cases and miRNA controls. Highlighted rows contain miRNAs of interest within the top three eigenmiRs. KEEG pathways were annotated to the target genes ^5-7^.

| Module |  | Eigenmirs |  | Top KEGG 2021 Pathway for eigenmiR’s targeted genes | Preservation State |
| --- | --- | --- | --- | --- | --- |
| blue | hsa-let-7g-5p | hsa-miR-26b-5p | hsa-let-7f-5p | FoxO signaling pathway | Preserved (72) |
| brown | hsa-miR-30e-5p | hsa-miR-191-5p | hsa-miR-199a-3p | Axon Guidance | New |
| green | hsa-miR-378a-3p | hsa-miR-378c | hsa-miR-378d | - | Preserved (29) |
| turquoise | hsa-miR-1268a | hsa-miR-642a-3p | hsa-miR-1268b | - | Preserved (79) |
| yellow | hsa-miR-186-5p | hsa-miR-146a-5p | hsa-miR-221-3p | Notch signalling pathway | Preserved (54) |

**Literature**

1 Umu, S. U. *et al.* A comprehensive profile of circulating RNAs in human serum. *RNA Biol* **15**, 242-250, doi:10.1080/15476286.2017.1403003 (2018).

2 Burton, J. *et al.* Serum RNA Profiling in the 10-Years Period Prior to Diagnosis of Testicular Germ Cell Tumor. *Front Oncol* **10**, 574977, doi:10.3389/fonc.2020.574977 (2020).

3 Robinson, M. D., McCarthy, D. J. & Smyth, G. K. edgeR: a Bioconductor package for differential expression analysis of digital gene expression data. *Bioinformatics* **26**, 139-140, doi:10.1093/bioinformatics/btp616 (2010).

4 Johnson, W. E., Li, C. & Rabinovic, A. Adjusting batch effects in microarray expression data using empirical Bayes methods. *Biostatistics* **8**, 118-127, doi:10.1093/biostatistics/kxj037 (2007).

5. Kanehisa, M. & Goto, S.; KEGG: Kyoto Encyclopedia of Genes and Genomes. *Nucleic* *Acids Res*. **28**, 27-30, doi.org/10.1093/nar/28.1.27 (2000)

6 Kanehisa, M; Toward understanding the origin and evolution of cellular organisms. *Protein Sci.* **28**, 1947-1951 doi.org/10.1002/pro.3715 (2019)

7. Kanehisa, M., Furumichi, M., Sato, Y., Kawashima, M. and Ishiguro-Watanabe, M.; KEGG for taxonomy-based analysis of pathways and genomes. *Nucleic Acids Res.* **51**, D587-D592, doi.org/10.1093/nar/gkac963 (2023)

**Supplementary table 3.** Mean gene expression for eigengenes (E1) related to cases (seminoma and non-seminoma subtypes together) vs control sample, with SD, minimum and maximum value as well as number of samples used. Genes are sorted alphabetically.

|  |  |  |
| --- | --- | --- |
| **Gene** | **All cases (seminoma and non-seminoma) Mean (SD, min-max, N)** | **Control Mean (SD, min-max, N)** |
| *BEST3* | 5.00 (7.56, 0.67-47.94, 80) | 5.00 (9.74, 0.73-76.03, 84) |
| *CPNE5* | 7.00 (9.20, 0.57-52.15, 80) | 6.00 (7.70, 0.67-42.32, 84) |
| *DNAJC17* | 7.00 (9.30, 0.54-53.89, 80) | 7.00 (8.49, 0.67-50.07, 84) |
| *GHRHR* | 4.00 (6.69, 0.58-41.88, 80) | 4.00 (3.57, 0.67-14.67, 84) |
| *KCNB1* | 28.00 (22.52, 1.10-116.27, 80) | 21.00 (18.21, 1.13-86.91, 84) |
| *KIAA2026* | 21.00 (15.02, 0.84-64.59, 80) | 20.00 (20.85, 0.95-160.38, 84) |
| *OPRD1* | 28.00 (23.18, 1.24-115.61, 80) | 23.00 (16.40, 2.15-82.08, 84) |
| *PCDHGB6* | 14.00 (11.00, 0.91-39.06, 80) | 12.00 (11.03, 1.00-45.65, 84) |
| *RTCB* | 18.00 (33.08, 1.01-262.13, 80) | 28.00 (121.54, 1.01-1105.50, 84) |
| *UBAC1* | 17.00 (13.13, 1.24-64.08, 80) | 17.00 (14.18, 1.27-56.26, 84) |
| *XPOT* | 8.00 (12.47, 0.91-84.54, 80) | 9.00 (10.48, 0.79-71.05, 84) |
|  |  |  |

**Supplementary table 4.** Mean gene expression for eigengenes (E1) related to seminoma vs non-seminoma samples, in addition to control samples with SD, minimum and maximum value as well as number of samples used. Genes are sorted alphabetically.

|  |  |  |  |
| --- | --- | --- | --- |
| **Gene** | **Seminoma** | **Non-Seminoma** | **Control** |
| *BEST3* | 5.00 (8.76, 0.67-47.94, 53) | 4.00 (4.31, 0.68-20.46, 27) | 5.00 (9.74, 0.73-76.03, 84) |
| *CFAP100* | 164.00 (630.45, 1.06-3743.60, 53) | 14.00 (15.77, 1.05-73.03, 27) | 28.00 (65.31, 0.91-468.40, 84) |
| *CHRNA7* | 16.00 (18.12, 1.13-72.86, 53) | 16.00 (12.78, 0.67-51.76, 27) | 14.00 (14.10, 0.98-66.01, 84) |
| *DPPA2* | 2.00 (1.05, 0.57-6.30, 53) | 2.00 (1.65, 0.58-8.23, 27) | 2.00 (1.79, 0.67-8.69, 84) |
| *FAM151B* | 5.00 (8.82, 0.54-37.95, 53) | 6.00 (7.28, 0.58-34.19, 27) | 4.00 (6.08, 0.67-43.59, 84) |
| *FASN* | 58.00 (66.50, 1.85-465.50, 53) | 39.00 (27.56, 3.33-117.02, 27) | 75.00 (113.65, 3.23-692.24, 84) |
| *GLIPR1* | 4.00 (6.36, 0.67-27.99, 53) | 5.00 (7.25, 0.68-28.94, 27) | 5.00 (7.21, 0.70-47.20, 84) |
| *HEPH* | 7.00 (7.32, 0.57-35.81, 53) | 7.00 (6.39, 0.68-26.82, 27) | 6.00 (5.76, 0.86-28.93, 84) |
| *NLRP11* | 5.00 (9.74, 0.67-57.32, 53) | 3.00 (3.76, 0.58-13.64, 27) | 4.00 (5.87, 0.67-27.58, 84) |
| *PANK3* | 13.00 (12.63, 0.54-47.99, 53) | 5.00 (5.81, 0.68-27.49, 27) | 10.00 (13.11, 0.98-76.70, 84) |
| *PDE6D* | 5.00 (8.56, 0.57-47.55, 53) | 6.00 (6.26, 0.67-30.41, 27) | 5.00 (8.58, 0.67-53.85 84) |
| *PPP1R13B* | 12.00 (11.66, 1.39-59.79, 53) | 11.00 (10.73, 0.68-48.52, 27) | 10.00 (9.02, 1.24-45.40, 84) |
| *RAB8B* | 8.00 (11.68, 0.54-65.72, 53) | 10.00 (14.53, 0.58-56.43, 27) | 7.00 (9.55, 0.67-54.46, 84) |
| *RP1-27O5.3* | 8.00 (9.09, 0.69-43.90, 53) | 8.00 (10.02, 0.68-43.76, 27) | 7.00 (8.30, 0.67-60.07, 84) |
| *RP11-729L2.2* | 1.00 (0.89, 0.67-5.57, 53) | 2.00 (2.73, 0.58-12.34, 27) | 2.00 (1.67, 0.70-10.06, 84) |
| *SCN5A* | 10.00 (10.32, 0.92-57.20, 53) | 10.00 (12.56, 1.08-59.32, 27) | 11.00 (14.91, 0.67-123.12, 84) |
| *SHC3* | 63.00 (44.23, 1.85-181.41, 53) | 47.00 (28.80, 3.32-113.12, 27) | 60.00 (178.51, 1.13-1642.84, 84) |
| *TBX5* | 4.00 (5.68, 0.54-32.30, 53) | 7.00 (11.76, 0.58-50.53, 27) | 6.00 (18.49, 0.67-168.67, 84) |
| *UBE2W* | 4.00 (7.43, 0.86-47.11, 53) | 4.00 (5.51, 0.88-27.26, 27) | 4.00 (5.31, 0.67-33.41, 84) |
| *USP14* | 10.00 (19.58, 0.67-129.00, 53) | 10.00 (19.52, 0.84-97.36, 27) | 8.00 (16.61, 0.79-138.04, 84) |
| *UVSSA* | 69.00 (78.59, 5.55-425.17, 53) | 41.00 (30.32, 3.33-139.85, 27) | 36.00 (33.91, 3.06-238.70, 84) |
| *ZC4H2* | 26.00 (21.38, 1.13-75.26, 53) | 22.00 (23.12, 1.23-102.75, 27) | 23.00 (22.27, 0.83-103.38, 84) |
|  |  |  |  |

**Supplementary table 5.** Mean gene expression for eigengenes (E1) related to seminoma vs non-seminoma samples, in addition to normal with SD, minimum and maximum value as well as number of samples. Genes are sorted alphabetically.

|  |  |  |  |
| --- | --- | --- | --- |
| **Gene** | **Seminoma** | **Non-Seminoma** | **Control** |
| *AAMDC* | 3.00 (5.33, 0.54-29.71, 53) | 2.00 (2.43, 0.83-13.56, 27) | 4.00 (8.98, 0.67-69.44, 84) |
| *ALMS1* | 26.00 (18.75, 1.04-75.33, 53) | 27.00 (27.20, 2.72-133.61, 27) | 27.00 (39.19, 0.79-340.36, 84) |
| *ANAPC4* | 8.00 (13.16, 0.58-61.11, 53) | 16.00 (37.77, 0.58-191.23, 27) | 8.00 (18.74, 0.73-162.10, 84) |
| *ARID4A* | 22.00 (20.71, 0.57-100.40, 53) | 18.00 (15.33, 1.07-53.17, 27) | 19.00 (15.69, 1.04-57.72, 84) |
| *C6orf118* | 2.00 (1.53, 0.54-11.03, 53) | 3.00 (5.10, 0.58-24.26, 27) | 2.00 (3.89, 0.67-34.63, 84) |
| *CBWD2* | 5.00 (9.31, 0.57-58.54, 53) | 5.00 (6.32, 0.83-27.11, 27) | 7.00 (8.14, 0.67-56.05, 84) |
| *CENPI* | 14.00 (21.57, 1.06-113.95, 53) | 11.00 (11.76, 0.88-44.24, 27) | 9.00 (13.72, 0.86-107.79, 84) |
| *CRAT* | 189.00 (275.55, 14.86-1377.37, 53) | 180.00 (363.08, 3.33-1747.82, 27) | 144.00 (238.69, 1.23-1215.94, 84) |
| *CROT* | 5.00 (10.50, 0.54-56.10, 53) | 6.00 (9.83, 0.68-36.97, 27) | 4.00 (6.18, 0.70-39.40, 84) |
| *DEK* | 17.00 (15.62, 0.81-60.69, 53) | 13.00 (11.86, 0.97-41.95, 27) | 16.00 (13.87, 1.09-86.97, 84) |
| *DNMT3A* | 33.00 (27.77, 1.85-129.61, 53) | 30.00 (17.41, 3.32-64.08, 27) | 27.00 (17.13, 1.48-83.47, 84) |
| *DPCD* | 5.00 (5.01, 1.05-24.70, 53) | 8.00 (10.30, 0.83-46.83, 27) | 8.00 (8.40, 0.86-33.07, 84) |
| *IL12RB1* | 7.00 (11.34, 0.57-55.92, 53) | 3.00 (3.79, 0.83-12.97, 27) | 6.00 (7.24, 0.70-39.30, 84) |
| *JAKMIP1* | 11.00 (10.53, 0.96-59.75, 53) | 14.00 (16.39, 1.22-60.77, 27) | 13.00 (12.72, 0.91-80.99, 84) |
| *LMBR1* | 29.00 (39.60, 1.22-265.63, 53) | 23.00 (17.01, 2.89-60.39, 27) | 26.00 (38.74, 1.16-253.71, 84) |
| *LZIC* | 11.00 (17.28, 0.81-91.59, 53) | 13.00 (16.68, 0.83-72.37, 27) | 10.00 (12.37, 0.77-76.04, 84) |
| *MAF* | 44.00 (40.00, 3.82-193.78, 53) | 31.00 (27.87, 2.46-121.80, 27) | 67.00 (172.92, 3.22-1340.38, 84) |
| *NREP* | 10.00 (11.56, 1.05-54.88, 53) | 9.00 (6.93, 1.03-24.29, 27) | 10.00 (8.17, 1.0-139.67, 84) |
| *OPRD1* | 26.00 (23.64, 1.24-115.62, 53) | 31.00 (22.27, 2.73-83.57, 27) | 23.00 (16.40, 2.15-82.08, 84) |
| *PELI1* | 152.00 (772.39, 0.58-5532.12, 53) | 13.00 (15.74, 0.83-64.91 27) | 30.00 (80.21, 0.67-577.36, 84) |
| *PPP1R1C* | 3.00 (5.94, 0.54-34.15, 53) | 3.00 (4.04, 0.58-18.87, 27) | 2.00 (2.31, 0.73-12.56, 84) |
| *PRKACB* | 3.00 (3.94, 0.54-22.25, 53) | 3.00 (6.25, 0.58-32.53, 27) | 4.00 (4.73, 0.67-18.79, 84) |
| *RABEPK* | 6.00 (13.58, 0.54-71.67, 53) | 8.00 (14.74, 0.69-59.28, 27) | 6.00 (11.75, 0.67-71.58, 84) |
| *SHISA5* | 10.00 (11.45, 0.58-56.88, 53) | 6.00 (7.04, 0.88-34.98, 27) | 7.00 (7.77, 0.67-47.69, 84) |
| *ULK2* | 11.00 (13.03, 0.86-57.58, 53) | 8.00 (7.48, 0.68-25.68, 27) | 10.00 (9.99, 0.98-43.71, 84) |
| *WAS* | 11.00 (14.23, 0.57-65.96, 53) | 7.00 (6.82, 0.84-24.79, 27) | 9.00 (10.10, 0.67-42.88, 84) |

**Supplementary table 6.** Mean gene expression for eigengenes (E1) related to seminoma vs non-seminoma samples, in addition to normal with SD, minimum and maximum value as well as number of samples. Genes are sorted alphabetically.

|  |  |  |  |
| --- | --- | --- | --- |
| **Gene** | **Seminoma** | **Non-Seminoma** | **Control** |
| *ATRX* | 40.00 (30.87, 2.62-171.81, 53) | 52.00 (47.42, 3.32-172.54, 27) | 40.00 (31.76, 3.22-238.55, 84) |
| *BEND7* | 11.00 (14.01, 1.15-86.58, 53) | 11.00 (11.93, 1.04-59.93, 27) | 17.00 (17.50, 1.01-82.92, 84) |
| *DCAF12* | 40.00 (48.00, 0.54-298.78, 53) | 23.00 (17.45, 1.02-57.92, 27) | 28.00 (37.05, 0.91-287.51, 84) |
| *FYCO1* | 8.00 (11.68, 0.58-58.66, 53) | 9.00 (8.44, 0.83-33.27, 27) | 8.00 (8.23, 0.84-41.46, 84) |
| *GMEB2* | 29.00 (38.12, 1.18-205.89, 53) | 16.00 (15.39, 1.42-73.94, 27) | 13.00 (13.45, 0.80-71.54, 84) |
| *GRM3* | 19.00 (24.86, 1.05-114.69, 53) | 22.00 (21.55, 0.83-75.63, 27) | 10.00 (12.76, 0.73-60.19, 84) |
| *IGLON5* | 5.00 (6.27, 0.86-25.36, 53) | 3.00 (2.53, 0.58-9.82, 27) | 3.00 (3.45, 0.66-15.48, 84) |
| *KLHL14* | 7.00 (8.74, 1.01-52.35, 53) | 7.00 (7.87, 0.90-28.64, 27) | 7.00 (13.59, 0.97-121.12, 84) |
| *MAP3K12* | 55.00 (63.95, 1.23-356.41, 53) | 246.00 (1089.71, 3.32-5697.10, 27) | 33.00 (28.62, 2.40-159.57, 84) |
| *MAPK4* | 5.00 (5.73, 0.69-25.92, 53) | 10.00 (9.89, 1.18-42.81, 27) | 8.00 (7.49, 1.00-36.24, 84) |
| *MASTL* | 12.00 (19.47, 0.92-104.88, 53) | 12.00 (14.97, 0.67-59.76, 27) | 9.00 (11.41, 0.73-76.70, 84) |
| *NRIP3* | 12.00 (19.72, 0.54-120.62, 53) | 13.00 (12.95, 0.87-44.46, 27) | 11.00 (11.83, 0.94-56.40, 84) |
| *PER2* | 6.00 (7.96, 0.57-41.37, 53) | 6.00 (8.10, 1.05-37.05, 27) | 6.00 (8.45, 0.73-47.06, 84) |
| *PPP1R37* | 18.00 (27.69, 1.05-199.29, 53) | 12.00 (12.94, 1.09-64.21, 27) | 13.00 (10.65, 1.19-53.59, 84) |
| *PRKCE* | 18.00 (17.86, 1.18-82.06, 53) | 19.00 (13.84, 1.09-49.56, 27) | 15.00 (15.17, 1.15-69.43, 84) |
| *SCN3A* | 16.00 (18.00, 0.53-72.32, 53) | 21.00 (36.20, 0.92-188.56, 27) | 14.00 (16.95, 1.01-93.30, 84) |
| *SLC25A36* | 23.00 (17.95, 1.53-70.44, 53) | 37.00 (52.72, 2.02-259.20, 27) | 19.00 (23.07, 1.28-166.96, 84) |
| *STYX* | 6.00 (11.22, 0.57-72.09, 53) | 4.00 (7.18, 0.58-35.47, 27) | 4.00 (5.04, 0.69-28.80, 84) |
| *TTLL2* | 8.00 (9.91, 1.06-41.17, 53) | 14.00 (18.80, 0.87-82.10, 27) | 9.00 (12.28, 0.78-82.36, 84) |
| *UCHL5* | 6.00 (9.61, 0.58-62.60, 53) | 4.00 (4.09, 0.68-14.82, 27) | 7.00 (9.86, 0.66-66.34, 84) |

**Supplementary figure 1:** Boxplots for PANK3 and TBX5 expression, contrasted with SOX17 expression (maker of seminoma) for seminoma, non-seminoma subtypes, and normal (raw expression, log10 scale).


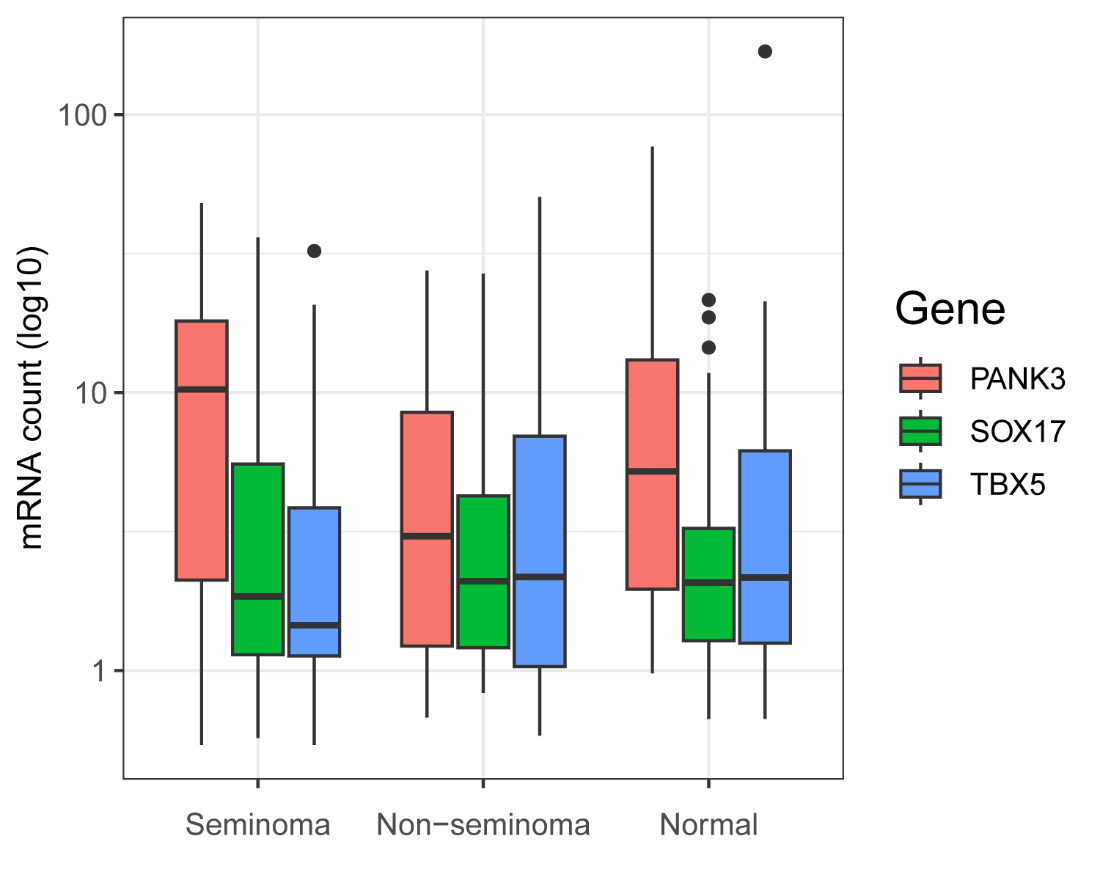


**Supplementary table 7**: Association between KIT (marker for seminoma) and eigengenes found specific to seminoma in our studies. Correlation coefficient, p-value of the association, a slope and an intercept from linear model added.

|  |  |  |  |  |  |
| --- | --- | --- | --- | --- | --- |
| **Gene vs** | **Gene** | **Correlation Coefficient** | **P-Value** | **Slope** | **Intercept** |
| *KIT* | *USP14* | -0.01 | 0.96 | -0.04 | 10.03 |
| *KIT* | *UBE2W* | -0.10 | 0.48 | -0.17 | 4.51 |
| *KIT* | *CFAP100* | -0.10 | 0.49 | -13.91 | 206.46 |
| *KIT* | *RP1-27O5.3* | 0.01 | 0.94 | 0.02 | 8.05 |
| *KIT* | *NLRP11* | -0.02 | 0.86 | -0.05 | 5.09 |
| *KIT* | *HEPH* | 0.10 | 0.48 | 0.17 | 6.92 |
| *KIT* | *DPPA2* | 0.04 | 0.78 | 0.01 | 1.70 |
| *KIT* | *GLIPR1* | -0.02 | 0.88 | -0.03 | 3.81 |
| *KIT* | *FAM151B* | -0.19 | 0.16 | -0.39 | 6.43 |
| *KIT* | *FASN* | -0.05 | 0.71 | -0.79 | 60.45 |
| *KIT* | *PPP1R13B* | -0.02 | 0.87 | -0.06 | 11.71 |
| *KIT* | *UVSSA* | 0.07 | 0.60 | 1.32 | 65.43 |
| *KIT* | *SCN5A* | -0.07 | 0.63 | -0.16 | 10.51 |
| *KIT* | *ZC4H2* | 0.08 | 0.55 | 0.41 | 24.68 |
| *KIT* | *TBX5* | 0.11 | 0.43 | 0.14 | 3.49 |
| *KIT* | *RP11-729L2.2* | -0.14 | 0.31 | -0.03 | 1.59 |
| *KIT* | *BEST3* | -0.08 | 0.57 | -0.16 | 5.68 |
| *KIT* | *RAB8B* | 0.04 | 0.79 | 0.10 | 8.01 |
| *KIT* | *SHC3* | -0.14 | 0.33 | -1.36 | 66.69 |
| *KIT* | *PDE6D* | -0.07 | 0.61 | -0.14 | 5.45 |
| *KIT* | *CHRNA7* | -0.07 | 0.64 | -0.27 | 17.14 |
| *KIT* | *PANK3* | -0.10 | 0.47 | -0.29 | 13.95 |

**Supplementary table 8:** Association between SOX17 (marker for seminoma) and eigengenes found specific to seminoma in our studies. Correlation coefficient, p-value of the association, a slope and an intercept from linear model added.

|  |  |  |  |  |  |
| --- | --- | --- | --- | --- | --- |
| **Gene vs** | **Gene** | **Correlation Coefficient** | **P-Value** | **Slope** | **Intercept** |
| *SOX17* | *USP14* | -0.11 | 0.43 | -0.32 | 11.46 |
| *SOX17* | *UBE2W* | -0.16 | 0.27 | -0.17 | 4.82 |
| *SOX17* | *CFAP100* | -0.02 | 0.88 | -1.92 | 173.48 |
| *SOX17* | *RP1-27O5.3* | -0.17 | 0.23 | -0.22 | 9.19 |
| *SOX17* | *NLRP11* | -0.06 | 0.64 | -0.09 | 5.37 |
| *SOX17* | *HEPH* | 0.00 | 0.98 | 0.00 | 7.43 |
| *SOX17* | *DPPA2* | -0.14 | 0.33 | -0.02 | 1.83 |
| *SOX17* | *GLIPR1* | 0.13 | 0.34 | 0.13 | 3.12 |
| *SOX17* | *FAM151B* | -0.13 | 0.36 | -0.17 | 6.06 |
| *SOX17* | *FASN* | 0.06 | 0.69 | 0.55 | 55.46 |
| *SOX17* | *PPP1R13B* | -0.21 | 0.12 | -0.37 | 13.29 |
| *SOX17* | *UVSSA* | 0.14 | 0.33 | 1.60 | 61.80 |
| *SOX17* | *SCN5A* | -0.06 | 0.69 | -0.09 | 10.45 |
| *SOX17* | *ZC4H2* | 0.04 | 0.77 | 0.13 | 25.29 |
| *SOX17* | *TBX5* | 0.33 | 0.02 | 0.28 | 2.60 |
| *SOX17* | *RP11-729L2.2* | -0.06 | 0.65 | -0.01 | 1.54 |
| *SOX17* | *BEST3* | -0.13 | 0.34 | -0.17 | 6.03 |
| *SOX17* | *RAB8B* | -0.17 | 0.22 | -0.29 | 9.71 |
| *SOX17* | *SHC3* | 0.21 | 0.13 | 1.38 | 56.02 |
| *SOX17* | *PDE6D* | -0.06 | 0.67 | -0.08 | 5.40 |
| *SOX17* | *CHRNA7* | -0.09 | 0.51 | -0.25 | 17.49 |
| *SOX17* | *PANK3* | -0.29 | 0.04 | -0.54 | 15.63 |
